# Arbuscular Mycorrhizal Fungi Increase Blast Resistance and Grain Yield in *Japonica* Rice Cultivars in Flooded Fields

**DOI:** 10.1186/s12284-025-00805-4

**Published:** 2025-05-30

**Authors:** Héctor Martín-Cardoso, Laia Castillo, Iratxe Busturia, Gerrit Bücker, Luís Marqués, Eva Pla, Mar Català-Forner, Concha Domingo, Blanca San Segundo

**Affiliations:** 1 Centre for Research in Agricultural Genomics (CRAG), CSIC-IRTA-UAB-UB, Campus Universitat Autònoma de Barcelona (UAB), CRAG Building, Bellaterra, 08193 Barcelona, C/ de la Vall Moronta Spain; 2 Cooperativa de Productores de Semillas de Arroz, S.C.L. (COPSEMAR), Avda del Mar 1, 46410 Sueca, Valencia Spain; 3 Sustainable Field Crops Centre d’Amposta, IRTA, 43870 Amposta, Catalonia Spain; 4 Departamento del Arroz and Centro de Genómica, Instituto Valenciano de Investigaciones Agrarias (IVIA), Ctra Moncada-Náquera km 10.7, 46113 Moncada, Valencia Spain; 5 Consejo Superior de Investigaciones Científicas (CSIC), Barcelona, Spain

**Keywords:** *Oryza sativa*, Arbuscular mycorrhiza, *Magnaporthe Oryzae*, Nursery, Paddy fields, Blast

## Abstract

Arbuscular mycorrhizal (AM) fungi establish symbiotic associations with a wide range of plant species. Root colonization by AM fungi improves the uptake of mineral nutrients in the host plant, mainly phosphorus, in exchange for photosynthetically fixed carbon. Rice is one of the most important cereal crops in the world that is cultivated in diverse ecosystems, mainly in flooded fields. Although rice is a host for AM fungi, flooding depresses colonization of rice roots by AM fungi. However, once fungal penetration into the rice root has occurred, the functional capacities of the AM fungus are not affected by flooding. In this study, we investigated mycorrhizal responsiveness in a panel of temperate *japonica* rice varieties in low fertility soil collected from rice fields. We show that inoculation with an AM fungus, either *Rhizophagus irregularis* or *Funneliformis mosseae*, stimulates seedling growth, improves Pi nutrition and enhances resistance to infection by the fungus *Magnaporthe oryzae* in aerobically grown rice plants in low fertility soil. The fungus *M. oryzae* is the causal agent of the rice blast disease, one of the most devastating diseases in cultivated rice worldwide. Field trials were conducted in flooded paddy fields of eastern Spain (mediterranean region) in 2023 and 2024. Three elite rice varieties were inoculated with *R. irregularis* and grown in nurseries under aerobic conditions during early vegetative stage. The AM-inoculated seedlings were then transplanted to flooded fields. We show that inoculation with *R. irregularis* increases grain yield and blast resistance, namely leaf blast, neck blast, node blast and panicle blast, in flooded field conditions. Although all the *japonica* rice varieties here examined benefited from the AM symbiosis, its effects varied depending on the rice variety and the geographical location. These findings demonstrated that the application of AM fungi in nurseries may be integrated with conventional rice cultivation systems in paddy fields for the development of sustainable rice production systems less dependent on chemical fertilizers and pesticides.

**Supplementary Information:**

The online version contains supplementary material available at 10.1186/s12284-025-00805-4.

## Key Message

The application of arbuscular mycorrhizal (AM) fungi in nurseries can be effectively combined with traditional rice cultivation practices in paddy fields, contributing to the establishment of sustainable rice production systems that are less reliant on fertilizers and pesticides.

**Supplementary Information:**

The online version contains supplementary material available at 10.1186/s12284-025-00805-4.

## Introduction

Rice (*Oryza sativa* L.) is one of the most important cereal crops in the world and serves as a staple food for more than half of the world’s population. The genus *Oryza* includes two cultivated species, *O. sativa* (Asian rice), which is further divided into *japonica* and *indica* subspecies, and *O. glaberrima* (African rice). While *japonica* rice varieties (e.g., temperate *japonica* rice) can be well adapted to temperate conditions, *indica* varieties are mainly cultivated in tropical areas. The most common system for rice cultivation is in flooded conditions (paddy fields), a system in which modern rice cultivars produce higher yields than in aerobic conditions. Growing rice in flooding fields, however, requires a vast amount of water. Therefore, rice cultivation faces challenges associated to climate change and water scarcity. Rice production is also seriously affected by diseases including the rice blast disease caused by the fungus *Magnaporthe oryzae* (Wilson and Talbot 2009). Treatments with chemical fungicides are routinely used to protect rice plants against blast, which causes serious environmental problems. The continuous decrease of arable land due to urbanization and industrialization, and eutrophication caused by indiscriminate use of agrochemicals also pose new threats to rice cultivation across the world.

Soil is a major source of nutrients needed by plants for growth and development. Regarding phosphorus (P) nutrition, plants acquire P primarily via the uptake of inorganic phosphate (Pi) through the roots. Even though the overall content of P in the soil is generally high, its low bioavailability represents a limiting factor for plant growth in many agricultural soils. Consequently, P fertilizers are routinely used to maintain optimal yield leading to excessive P accumulation in soils and environmental concerns. Additionally, P is a limited resource, and the high agricultural P demand has put the sustainability of P mining for fertilizer production into question. In order to ensure food security, it is urgent to develop more productive rice cultivation systems, less dependent on the use of agrochemicals (e.g., fertilizers and pesticides).

The use of natural biostimulants such as arbuscular mycorrhizal fungi, offers valuable alternatives to ensure crop yields in a sustainable manner. Arbuscular mycorrhizal (AM) fungi are commonly found in all earth’s ecosystems with plants and are considered natural biofertilizers. They can colonise the roots of over 90% of terrestrial higher plants to the mutual benefit of both the plant host and the fungus (Parniske 2008; Choi et al. 2018; Genre et al. 2020). Root colonization by AM fungi improves nutrient and water in the host plant, mainly Pi, in exchange for photosynthetically fixed carbon. Besides improving plant nutrition, the AM symbiosis can enhance plant resistance against pathogen infection and/or tolerance to abiotic stress (Ruiz-Sánchez et al. 2010; Fiorilli et al. 2011; Jung et al. 2012; Eke et al. 2019; Campo et al. 2020; Pozo de la Hoz et al.^ 2021^). Mycorrhiza-induced resistance (MIR) has been shown to be associated with priming of plant defense responses (Pozo and Azcón-Aguilar 2007; Jung et al. 2012). Most of our knowledge on the establishment and functioning of the AM symbiosis comes from studies in the model legumes *Medicago truncatula* and *Lotus japonicus* and more recently also in horticultural crop species (e.g., *Solanum lycopersicum*).

Most studies to assess the impact of the AM symbiosis in rice have been carried out on plants in soilless cultivation using inert substrates to grow plants in greenhouses (e.g., sand, turface and vermiculite mixes) under aerobic conditions. Although these systems allow a better control of nutrient supply, their use raises the question of whether the properties of those substrates might affect Pi acquisition efficiency in the host plant, hence, its susceptibility to be colonized by the AM fungus. Here, it is important to consider that, in plants grown on inert substrate, nutrients are supplied through fertigation, as compared to plants grown on natural soil where the physical and chemical soil properties of the soil might affect nutrient availability and root colonization by AM fungi. Additionally, in soil-grown plants, AM fungi might work together with other soil microorganisms to improve plant nutrition, such as Plant Growth-Promoting Bacteria (PGPB) or Phosphate Solubilizing Bacteria (Etesami et al. 2021). The presence of microorganisms in soil also depends on its properties, chemical composition, pH and structure. Clearly, the symbiotic association between plants and AM fungi evolved to help the plants to acquire external P, particularly when soil P levels are low (Balzergue et al. 2013). Along with this, the response of plants to AM colonization in natural soils, and the capacity to benefit from the AM symbiosis might differ from that in soilless culture systems.

Natural root colonization by AM fungi in rice plants grown under aerobic conditions has long been demonstrated (Vallino et al. 2009; Bernaola et al. 2018). However, the AM symbiosis in rice has been poorly explored, most probably because for many years plants grown under waterlogging conditions, were considered not to be susceptible of colonization by AM fungi. Nowadays, it is generally accepted that rice plants are capable of establishing symbiotic associations with AM fungi, also under flooded conditions (Wang et al. 2021). Natural occurrence of AM fungi in rice paddy fields, including the well-studied *Rhizophagus irregularis* and *Funneliformis mosseae*, is documented (Lumini et al. 2011; Wang et al. 2015, 2021; Chialva et al. 2020; Surendirakumar et al. 2021; Barro et al. 2022). It is also known that although growing rice under permanent flooded conditions reduces root colonization by AM fungi in rice, once the fungus has penetrated the root system, the functional capacities of the AM fungus are not affected by flooding (Vallino et al. 2014; Wang et al. 2021). Very recently, AM fungi have been shown to mediate the acclimation of rice to submergence (Xu et al. 2024). Knowing that rice establish associations with AM fungi, it is of vital importance to further investigate the benefits that rice plants can receive from the AM symbiosis in agricultural ecosystems. Previous studies in aerobically grown rice (cv. Senia) under controlled conditions demonstrated that inoculation with the AM fungus *R. irregularis* promotes the induction of defense-related genes in leaves and enhances resistance to infection by *M. oryzae* (Campos-Soriano et al. 2012). Varietal differences in the response of mycorrhizal rice plants to infection by the rice blast fungus have also been observed in plants grown on inert substrates, in which positive, neutral and negative responses to *M. oryzae* infection were found to occur (Campo et al. 2020; Guigard et al. 2023).

A major impediment to exploiting the AM symbiosis in rice cultivation is that modern rice varieties have been developed based on the use of hight inputs of P fertilizers. Plants and AM fungi are expected to adapt to each other in their environment, and mycorrhizal associations are usually reduced upon sustained application of P fertilizers in the field. Additionally, modern rice cultivars are the product of breeding and selection processes typically performed under high fertilization conditions which might have resulted in selection of rice genotypes with less capability to establish associations with AM fungi. Therefore, the high input of P fertilizers might have weakened the association between modern rice cultivars and AM fungi. From the perspective of practical application, more research efforts are required to assess the effects of AM fungi in modern elite rice varieties well adapted to specific agro-climatic conditions under field conditions.

In this work, we evaluated mycorrhizal responsiveness in a panel of temperate *japonica* rice varieties cultivated in the mediterranean rice-producing regions (eastern Spain). This study was performed on 9 *japonica* rice varieties grown on low fertility soil collected from rice fields. Based on the above indicated considerations on root colonization by AM fungi in plants grown under aerobic or flooded conditions, we reasoned that growing AM-inoculated rice seedlings on aerobic conditions (nursery stage) during early growth would allow penetration of the AM fungus into the rice roots, and then transplanting the AM-inoculated rice seedlings to paddy fields would allow development of a functional symbiosis. In this direction, the mycorrhizal responsiveness of the 9 rice varieties was initially examined in nursery seedlings in low fertility soil using two different AM fungi, *Rhizophagus irregularis* and *Funneliformis mosseae*. Plant growth, Pi content and blast resistance was evaluated in the panel of rice cultivars inoculated with one or another fungus. Next, field assays were conducted on three selected rice cultivars, Albufera, Bomba, JSendra. Blast resistance and productivity was determined in AM-inoculated and mock-inoculated plants under natural infection conditions in 2 consecutive years (2023, 2024) in 2 geographical regions (Valencia and Ebro river delta). Our data provide clues to enhance yield and disease resistance in commercial rice cultivars while reducing the use of agrochemicals (fertilizers, pesticides) in rice fields.

## Materials and Methods

### Plant and Soil Materials

Commercial rice cultivars (*O. sativa* cv. *japonica*) assayed in this study were: Albufera, Argila, Baixet, Bomba, Copsemar 8, Guara, JSendra, Marisma and Regina. These cultivars are well adapted to temperate climate conditions and widely cultivated in rice-growing regions in Spain.

Natural soils used to grow the rice plants were collected from rice fields located at the Ebro river delta, a traditional rice producing region. Soil from a rice field that has not received any fertilizer for the last 25 years (from now on referred to as low fertility soil) was used. For comparative purposes, in some experiments, we used soils collected from fields which has been over-fertilized for more than 5 years (or high-fertility soil). The location of fields from which soils were collected is shown in Fig. S1. Basic properties and nutrient content from these soils are shown in Table 1. Soil analysis was carried out by spectrophotometry UV-VIS for Phosphorus and Nitrogen, Spectrophotometry ICP-OES for potassium and calcium, potentiometric titration for calcium carbonate and oxidizable organic matter, and potentiometry for pH (Eurofins Agroenvironmental, https://www.eurofins.es/). Soils were not sterilized to avoid elimination of the indigenous microbial population.

**Table 1:** Properties of soil used to grow rice cultivars

Nutrient content and properties	Low fertility soil	High fertility soil
Phosphorus (Olsen method)	21.8 mg/Kg*	94.0 mg/Kg*
Nitrogen	8.2 mg/Kg*	7.6 mg/Kg*
Potassium	100 mg/Kg*	206 mg/Kg*
Calcium	6791 mg/Kg*	7405 mg/Kg*
Calcium carbonate	37.41%	34.14%
Electrical conductivity (25ºC)	1.0 dS/m	0.48 dS/m
pH (1:2,5, soil: water suspension)	8.2	8.0
Oxidizable organic matter	1,42%*	3.82%*
Texture	Clay loam ^1^	Loam ^2^

Soils were collected from rice fields located in the rice growing region of the Ebro river delta (Spain), both low fertility and high fertility soils. Location of the rice fields in the Ebro river delta can be seen in Fig. S1. Low-fertility soil was used in mycorrhization experiments. *, Calculated on dry matter. ^1^ Texture USDA Bouyoucos: Clay (˂ 0.002 mm), 29.8%; Silt (0.002–0.05 mm), 47.0%; Sand (0.05–2 mm), 23.1%. ^2^ Texture USDA Bouyoucos: Clay (˂ 0.002 mm), 25.8%; Silt (0.002–0.05 mm), 33.1%; Sand (0.05–2 mm), 41.1%

## Inoculation with AM Fungi and Growth of Rice Plants in Nursery

Rice seeds were dehusked, surface-sterilized with ethanol 70% (3 min), sodium hypochlorite (6%, containing 0.02% Tween 20; 30 min) and thoroughly washed with sterile water with gentle shaking. Seeds were then germinated on petri dishes with sterile water to ensure that all seeds germinated (10 days at 28ºC/25ºC, 12 h/12 h light/dark cycle). Two AM fungi were assayed, *Rhizophagus irregularis* (formerly *Glomus intraradices*; *Rhizophagus intraradices*; FR121) and *Funneliformis mosseae* (formerly *Glomus mosseae*; FR140). Commercial inocula of AM fungi were purchased from MycAgro (Granular AMF Inoculum; Bretenière, France; http://www.mycagrolab.com). The inoculum consists of mineral inert solid particles and propagules of AM fungi (e.g., spores, mycelium and mycorrhizal root pieces) at a concentration of 10 propagules/g dry mass. For inoculation of rice seedlings with an AM fungus, the commercial inoculum was mixed with the plant growth substrate at a ratio of 5% v/v (AM inoculum/substrate). The rice plants were grown on seedling beds (35 cell trays; 350 mL each cell) filled with the AM/soil mixture. No inoculum was added to the substrate for the control plants (referred to as mock-inoculated). Then, AM-inoculated and mock-inoculated seedlings were aerobically grown in a nursery for 5 weeks at 28ºC/25ºC, 14 h/10 h light/dark cycle and high humidity, and then transplanted into flooded fields (Fig. S2A). During this period, the rice seedlings were supplied with modified Hoagland’s solution with low-Pi concentration (25 µM KH_2_PO_4_) every 2 days (10 mL/cell). Measurements of plant height were carried out by image analysis on pictures taken at 5 weeks post inoculation with an AM fungus, or mock-inoculation, using the ImageJ/Fiji v1.54j software (http://fiji.sc/Fiji).

## Pi Content

The rice plants were inoculated with an AM fungus and grown in the nursery under aerobic conditions as described above (Fig S2A). Pi content was determined on the youngest totally expanded leaves at 5 weeks after inoculation with the AM fungus. Three biological replicates were analyzed, each one consisting of a pool of three leaves obtained from three individual plants. Pi content of rice leaves was determined using a colorimetric method as previously described (Ames 1966; Versaw and Harrison 2002). Three independent experiments were carried out with similar results.

## Blast Resistance Assays Under Controlled Conditions

The fungus *M. oryzae* (strain Guy-11) was grown on Complete Media Agar (CMA) supplemented with chloramphenicol (30 mg/L) for 15 days at 25 °C 14 h/10 h light/dark photoperiod. *M. oryzae* spores were collected by adding sterile water to the mycelium surface. Spore suspensions were filtered, microscopically examined and adjusted to the desired concentration. Rice seedlings at the 3–4 leaf stage (e.g., 3 to 4- week-old plants) were sprayed with a suspension of *M. oryzae* spores (5 × 10^5^ spores/mL; 0.4 mL/plant) using an aerograph at 2 atm of pressure (Escolà et al. 2023) (Fig. S2B). Control plants were inoculated with sterile water. The inoculated seedlings were maintained in the dark overnight (at 90% humidity) and allowed to continue growth under a 14 h/10 h light/dark cycle under controlled conditions. Progression of disease symptoms was followed over time. The percentage of leaf area affected by blast lesions was determined at 7 days post-inoculation with *M. oryzae* spores using the APS Assess 2.0 program (Lamari 2008). Three independent experiments were carried out with similar results.

For quantification of fungal biomass, genomic DNA was extracted from *M. oryzae-*inoculated leaves as described above (Murray and Thompson 1980). Quantitative PCR (qPCR) was carried out using specific primers for the *M. oryzae* 28S DNA gene (forward, 5’-TACGAGAGGAACCGCTCATTCAGATAATTA-3’; reverse, 5’-TCAGCAGATCGTAACGATAAAGCTACTC − 3’), and normalized to the rice *Ubiquitin 1* gene (*OsUbi1*, Os06g0681400) (forward, 5’-TTCCCCAATGGAGCT-3’; reverse, 5’-AAACGGGACACGACCAAGG-3’) (Qi and Yang 2002).

## Analysis of Root Colonization

Root colonization by the AM fungus was assessed by microscopic observations of cotton blue-stained roots (0.1% cotton blue in acid lactic) as described (Berruti et al. 2013). For this, pre-germinated seedlings were transplanted to 160 mL-cones (21 cm in length; 160 mL each cone) containing inert substrate (66.7% quartz sand, 0.3–0.8 mm; 22.2% turface; 11.1% vermiculite), with or without the AM inoculum, and supplied with modified Hoagland’s solution with low-Pi concentration (10 mL/cone) as previously described (Campo et al. 2020). Root colonization was assessed at 5 weeks and 10 weeks after AM inoculation (Fig. S2C). The rice roots were extensively washed with sterile water, cut into 1 cm fragments and mounted onto microscope slides. Estimation of mycorrhizal colonization in roots fragments (25 root fragments) was carried out according to the Trouvelot method (Trouvelot 1986). The frequency of colonization was calculated as the number of colonized root pieces to the total number of visualized root pieces, in three independent experiments. Fungal structures were visualized on a Leica DM6 microscope under bright field illumination.

The molecular detection of *R. irregularis* and *F. mosseae* in AM-inoculated rice roots was also approached in this work. For this, DNA was extracted from mock and AM-inoculated rice roots as previously described (Murray and Thompson 1980) but using MATAB (0.1 M Tris-HCl pH 8.0, 1.4 M NaCl, 20 mM EDTA, 2% MATAB, 1% PEG 6000 and 0.5% Na_2_SO_3_) as the extraction buffer. AM fungi were detected using the polymerase chain reaction (PCR) using specific primers for the 28S rRNA gene of *R. irregularis* (forward, 5’-TTCGGGTAATCAGCCTTTCG-3’; reverse, 5’-TCAGAGATCAGACAGGTAGCC-3’) and the small subunit rRNA gene of *F. mosseae* (forward, 5’-GGAAACGATTGAAGTCAGTCATACCAA-3’; reverse, 5’- CGAAAAAGTACACCAAGAGATCCCAAT-3’) (Thonar et al. 2012). The PCR reaction consisted of: 2 µL Buffer 10X (Agilent, USA), 0.4 µL of dNTPs (GeneCraft, Germany) (from a 10 µM stock solution), 0.4 µL each primer (from a 10 µM stock solution), 0.4 µL MgCl_2_ (from a 25mM stock solution), 14.2 µL ddH_2_O, 0.2 µL Paq polymerase (Agilent, USA) and 2 µL DNA, in a total volume of 20 µl. The PCR products (10µL) were examined on a 1% agarose gel and their nucleotide sequence was confirmed by DNA sequencing. The percentage of sequence homology was 97% with the *R. irregularis* genomic DNA (HF968934.1; strain DAOM229456; https://www.ncbi.nlm.nih.gov/nuccore/HF968934.1?report=genbanksequence) and 95% with the *F. mosseae* genomic DNA (MK937794.1 https://www.ncbi.nlm.nih.gov/nuccore/MK937794.1?report=genbank).

### Field Trials

**Fig. 1:**
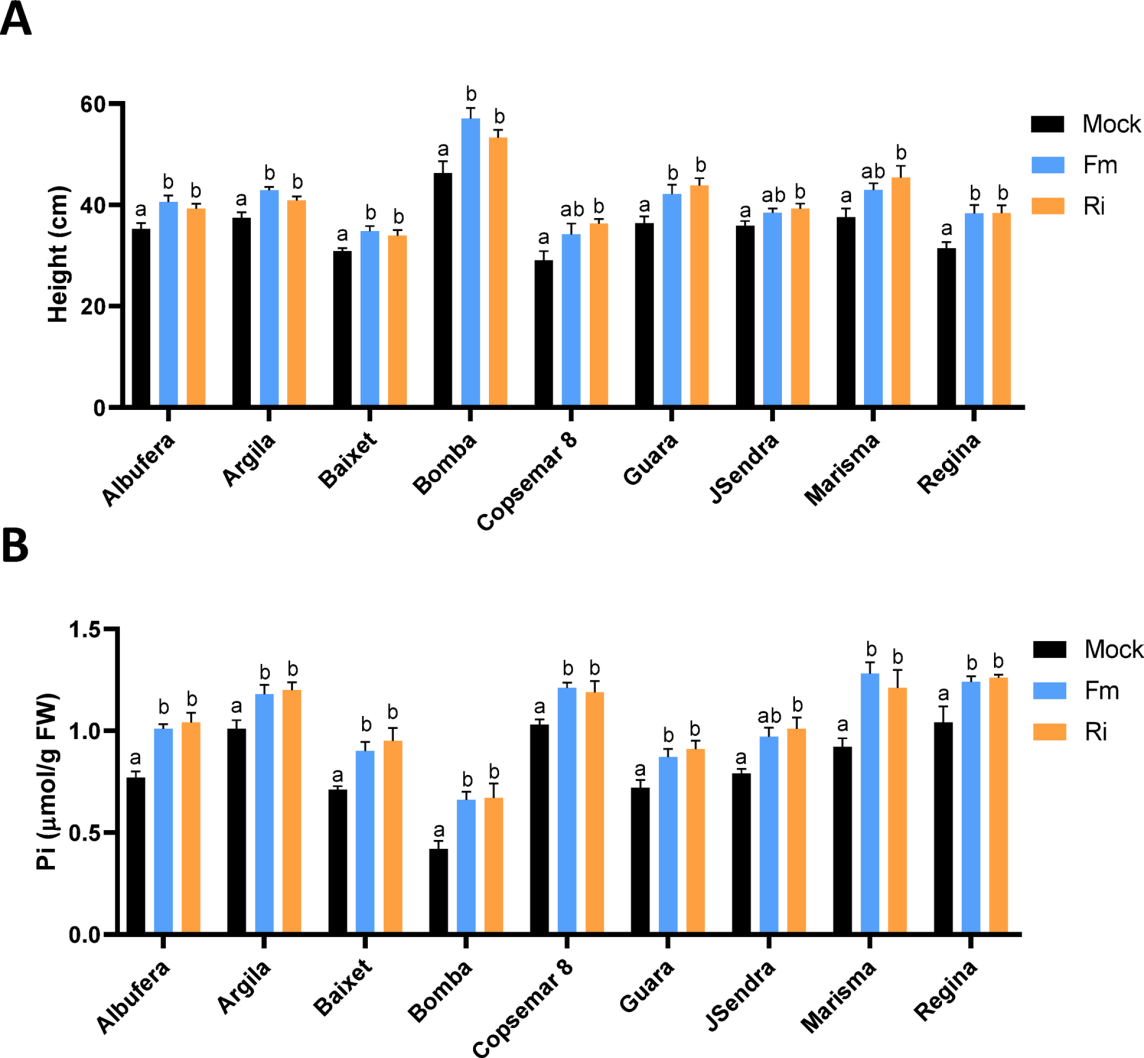
Growth and leaf Pi content in AM-inoculated rice cultivars grown in natural low fertility soil. Measurements were made in rice plants at 5 weeks post-inoculation with *R. irregularis* (Ri), *F. mosseae* (Fm), or mock-inoculation (mock). Statistically significant differences were determined by one-way ANOVA with Tukey’s multiple comparisons post hoc test (different letters indicate significant differences between treatments; P adjusted value < 0.05). **A** Shoot height of rice plants. Bars represent mean ± SEM of 9 biological replicates. **B** Pi content in leaves of rice plants. Bars represent mean ± SEM of 3 biological replicates, each one from a pool of 3 plants. Three independent experiments were carried out with similar results

Field experiments were conducted in two rice-growing regions in eastern Spain, namely Valencia (39˚15’46.9”N 0˚20’41.2”W) and Ebro river delta (40°42’26.2"N 0°37’57.8"E), in two successive years (2023 and 2024). Field experiment location can be seen in Fig. 1. Effects of inoculation with *R. irregularis* on blast resistance and grain yield were examined in three widely cultivated *japonica* rice varieties in these geographical regions, Albufera, Bomba, and JSendra.

Rice seedlings were inoculated with *R. irregularis*, or mock-inoculated, grown in the nursery under aerobic conditions for 5 weeks, and manually transplanted into flooded rice fields. The rice plants were allowed to grow until maturity (May-September) (Fig. S2A). Neither fertilizers nor pesticides were applied to rice plants in the field. A random block design was used for field trials, each with 3 replications (plots) for each variety (Albufera, Bomba, JSendra) and condition (*R. irregularis*- and mock-inoculated) (Fig. S3). Each elementary plot consisted of 30 plants (each genotype and condition). Blast resistance in field-grown plants was assessed at the harvest time in the two rice-growing regions under natural infection conditions, attending to leaf blast, neck blast, node blast, and panicle blast.

Leaf blast resistance was assessed using the Standard Evaluation System (SES) from the International Rice Research Institute (IRRI) (http://www.knowledgebank.irri.org). The SES score for leaf blast rates the symptoms of *M. oryzae* infection on a scale of 1 to 9, where a lower score of SES indicates resistance and a higher score indicates susceptibility to *M. oryzae* infection. Neck blast was determined by counting the number of panicles with neck blast in 40 randomly harvested samples from each plot (expressed as percent neck blast). Node blast was determined by recording node symptoms in 6 plants randomly harvested from each plot (expressed as percent node blast). Incidence of panicle blast was determined on 40 randomly harvested panicles and expressed as the percentage of panicles showing at least 25% of damage.

For evaluation of grain yield, grains from all the plants in each individual plot (for each variety and condition) were collected, pooled and weighted. The number of panicles per plant was also recorded for each individual plot.

Germinability (capacity to germinate) of seeds harvested from field trials in 2024 (mycorrhizal and non-mycorrhizal plants) was examined. For this, the seeds were sterilized as described above and germinated on water (28ºC/25ºC, 12 h/12 h light/dark cycle). Percent germination during the time interval of 4 days was calculated and expressed as the number of seeds with radicles at least 4 mm long (3 technical replicates, *N* = 25 each).

### Statistical Analysis

The Student’s *t*-test was used for measurements of Pi content in low and high fertility soil, blast analysis in field assays and productivity and germination. The thresholds for significance were as follows: *P* < 0.05 (*), *P* < 0.01 (**) and *P* < 0.001 (***). One-way ANOVA was used to estimate height, Pi content, lesion area and fungal biomass of plants grown in low fertility soil. One way ANOVA analysis was followed by Tukey’s multiple comparisons post hoc test (P adjusted value < 0.05).

## Results

### Improved Growth in AM-inoculated *japonica* Rice Cultivars in Low Fertility Soil

Since mycorrhizal function is to improve Pi acquisition under Pi scarcity, one potential challenge to implement the use of AM fungi in rice cultivation is that high soil P concentrations might hamper root colonization by AM fungi. It is then necessary to discern whether natural soils from rice fields favour rice/AM fungus interactions. Accordingly, we evaluated growth and performance of rice varieties on soils collected from rice fields with no fertilizer application for 25 years (henceforth low fertility soil). The available phosphorus (Olsen-Pi) level was 21.8 mg/Kg, the low fertility soil being also moderately alkaline (pH 8.2) (Table 1). In this respect, the availability of Pi to plants is known to be reduced in moderately alkaline soils (i.e., typically greater than 7.0 to 9.0) due to the formation of poorly soluble calcium phosphate minerals. Other properties of the low fertility soil used in this study are presented in Table 1. For comparative purposes, rice plants were grown in a highly fertilized soil (henceforth high fertility soil) whose P Olsen value was 4.3-fold higher than that in the low fertility soil (94.0 mg/Kg Olsen P) (Table 1). Compared to low fertility soil, the high fertility soil also had a higher content of organic matter (e.g., 2.7-fold higher) (Table 1). Soil organic matter acts as a source of Pi in the soil making Pi more available to plants.

Responsiveness to inoculation with an AM fungus was assessed in the panel of *japonica* rice cultivars grown on natural low fertility soil. The following rice cultivars were used in this study: Albufera, Argila, Baixet, Bomba, Copsemar 8, Guara, JSendra, Marisma and Regina. The phenotypic response to inoculation by an AM fungus, *Rhizophagus irregularis* and *Funneliformis mosseae* was assessed. Measurements of shoot height revealed that inoculation with one or another AM fungus stimulated plant growth in all rice varieties (Fig. 1A). Growth stimulation in AM-inoculated plants correlated with an increase in leaf Pi content in these plants compared to the corresponding mock-inoculated plants (Fig. 1B). Compared with plants grown in low fertility soil (see Fig. 1), growing rice cultivars in highly fertilized soil was accompanied by an increase in leaf Pi content (Fig. S4), thus, supporting a link between plant growth and the Pi status of the plant. Together, these findings demonstrated that inoculation with an AM fungus improves Pi nutrition and effectively promotes the growth of *japonica* rice varieties in natural, low fertility soils. Beneficial effects of AM inoculation were observed with both AM fungi, *R. irregularis* and *F. mosseae.*

### Enhanced Resistance to Infection by ***M. oryzae*** in AM-Inoculated Rice Plants in Low Fertility Soil

Many studies have been carried out to evaluate disease resistance on mycorrhizal plants, most of them using inert substrates to grow the plants (e.g., soilless media used in greenhouse cultivation). Variable responses to infection by the rice blast fungus *M. oryzae* were previously observed in rice plants grown on quartz sand/turface/vermiculite mixtures that have been inoculated with an AM fungus (Campo et al. 2020). In such conditions, positive, neutral, and even negative effects on blast disease could be observed in AM-inoculated plants, a response that was dependent on the rice variety (Campo et al. 2020).

Resistance to infection by the rice blast fungus was investigated in the panel of rice varieties inoculated with an AM fungus, either *R. irregularis* or *F. mosseae*, growing on natural, low fertility soil. As controls, seedlings were mock-inoculated. At 4 weeks after AM inoculation, the plants were sprayed with a suspension of *M. oryzae* spores, and development of disease symptoms was followed over time.

**Fig. 2:**
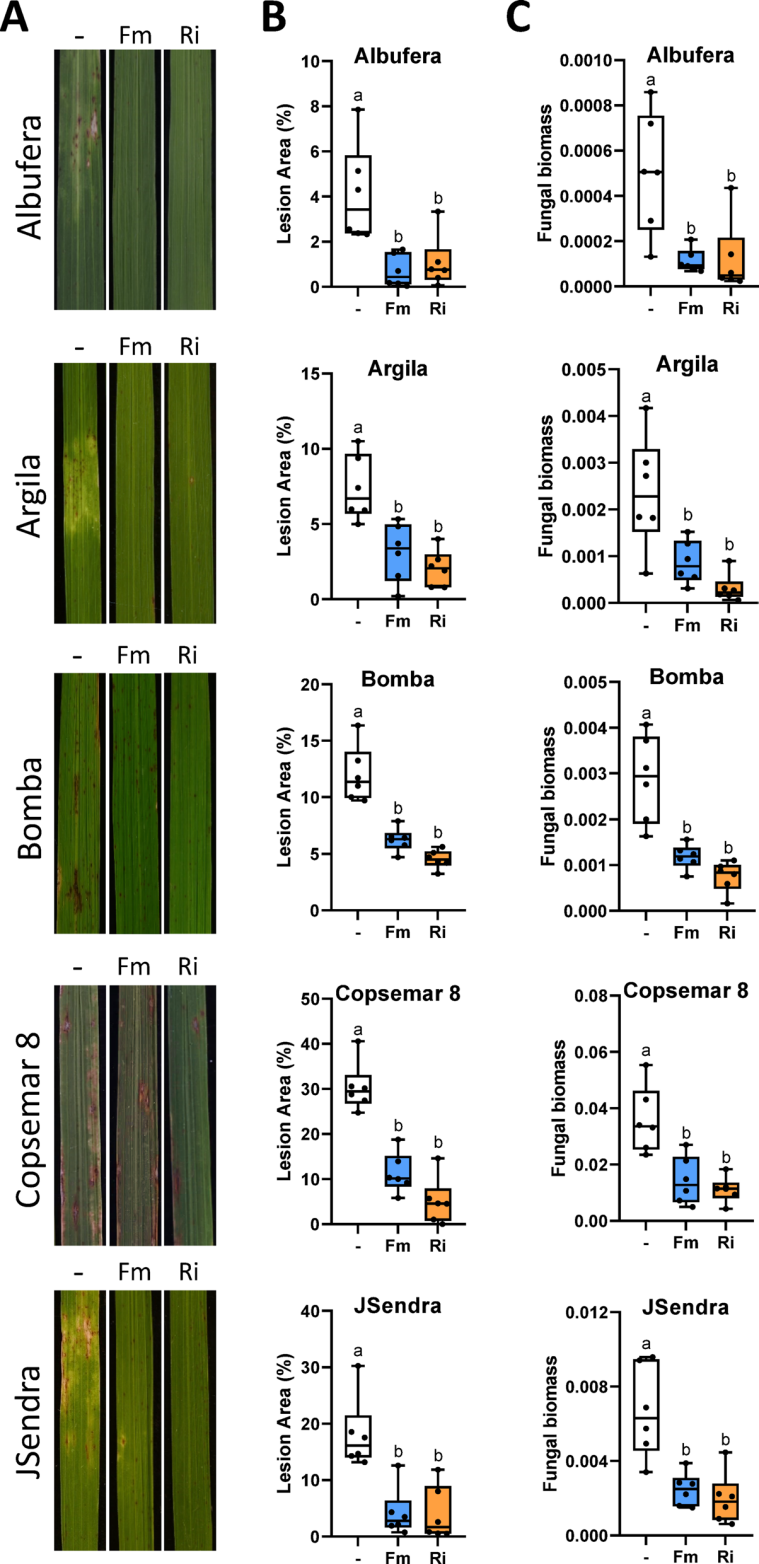
Blast resistance in rice cultivars grown in low fertility soil. Seedlings of Albufera, Argila, Bomba, Copsemar 8 and JSendra rice cultivars were grown in low fertility soil collected from rice fields. Seedlings were inoculated with the AM fungus *R. irregularis* (Ri), *F. mosseae* (Fm) or mock-inoculated (-). Four weeks after inoculation with the AM fungus, the rice plants were infected with *M. oryzae* spores. Blast resistance was assessed at 7 days after *M. oryzae* infection. Three independent experiments were carried out with similar results. Boxplot represents median and data distribution (*N* = 6). Statistically significant differences were determined by one-way ANOVA with Tukey’s multiple comparisons post hoc test (different letters indicate significant differences between treatments; P adjusted value < 0.05. **A** Blast phenotype in rice leaves. Representative images are shown. **B** Quantified blast lesion area in rice leaves by APS Assess 2.0 software. **C***M. oryzae* fungal biomass in rice leaves

**Fig. 3:**
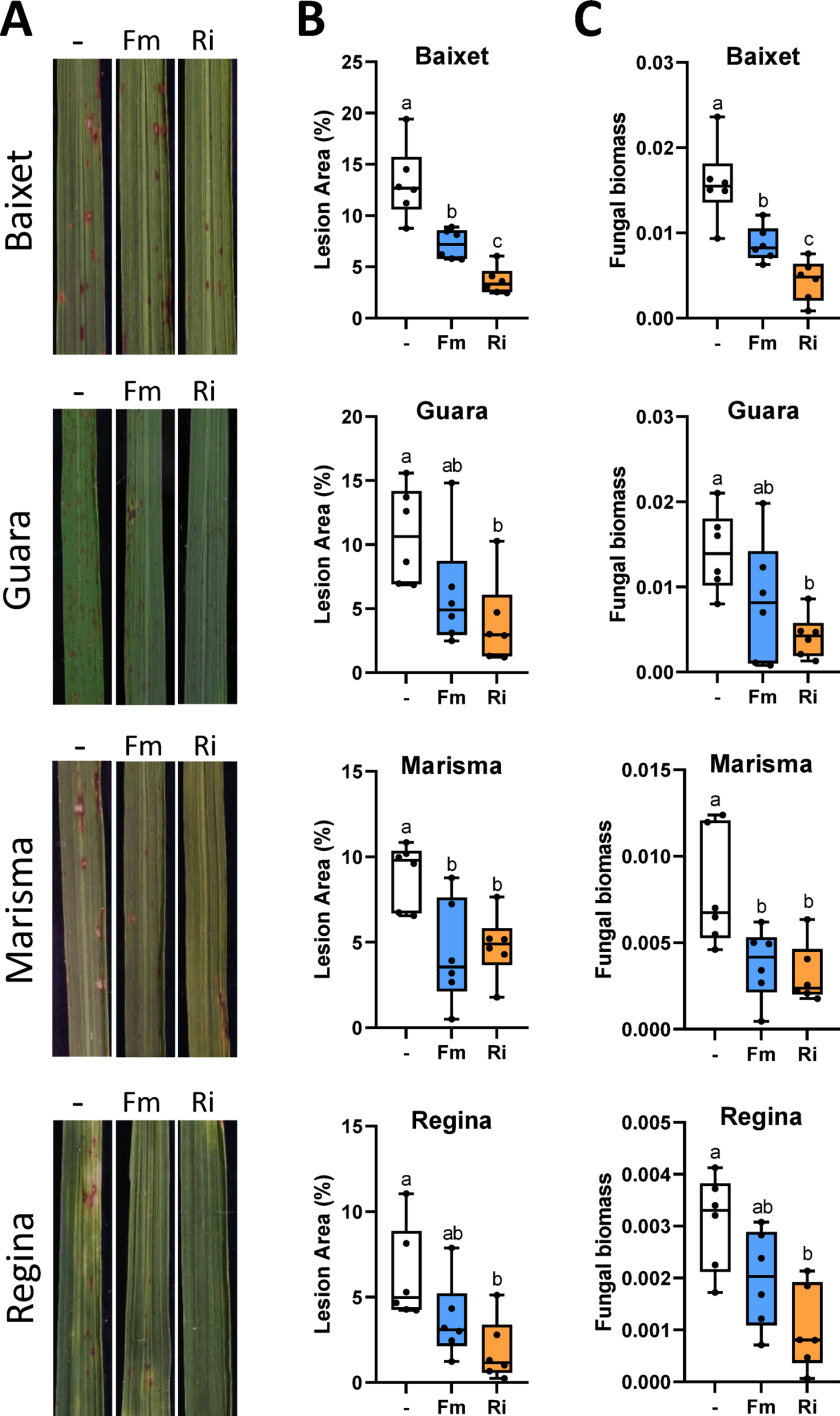
Blast resistance in rice cultivars grown in low fertility soil. Seedlings of Baixet, Guara, Marisma and Regina rice cultivars were grown in low fertility soil collected from rice fields. Seedlings were inoculated with the AM fungus *R. irregularis* (Ri), *F. mosseae* (Fm), or mock-inoculated (-). Three weeks after inoculation with the AM fungus, the rice plants were infected with *M. oryzae* spores. Blast resistance was assessed at 7 days after *M. oryzae* infection. Three independent experiments were carried out with similar results. Boxplot represents median and data distribution (*N* = 6). Statistically significant differences were determined by one-way ANOVA with Tukey’s multiple comparisons post hoc test (different letters indicate significant differences between treatments; P adjusted value < 0.05). **A** Blast phenotype in rice leaves. Representative images are shown. **B** Quantified blast lesion area in rice leaves by APS Assess 2.0 software. **C***M. oryzae* fungal biomass in rice leaves

Visual inspection of *M. oryzae*-infected plants revealed reduced blast symptoms in AM-inoculated rice plants compared to their mock-inoculated counterparts (Figs. 2 and 3). Blast resistance in AM-inoculated plants was confirmed by quantifying the leaf area with lesions and measuring the amount of fungal DNA, an indicator of fungal biomass in the plant tissue (Figs. 2 and 3). No negative effects could be observed on blast resistance in any of the AM-inoculated *japonica* rice cultivars investigated in this study.

Altogether, this study demonstrated that inoculation with an AM fungus, either *R. irregularis* or *F. mosseae*, enhances blast resistance in *japonica* rice cultivars in low fertility soil from rice fields.

### Root Colonization by AM Fungi in Rice Nursery Seedlings

Most rice varieties grow best in flooded fields, which makes rice one of the world’s most water-demanding crop. In previous studies, it was reported that growing rice under conventional flooding negatively impacts root penetration by AM fungi (Lumini et al. 2011). Later on, it was found that, once the fungus penetrated the roots, fungal viability in not affected by flooding hence allowing functionality of the AM symbiosis in flooded conditions (Vallino et al. 2014; Wang et al. 2021). With this in mind, we tested whether the rice plant can receive benefits from the AM symbiosis by first growing the AM-inoculated seedlings aerobically in nurseries (first weeks of vegetative growth) and then transplanting them into flooded rice fields.

**Fig. 4:**
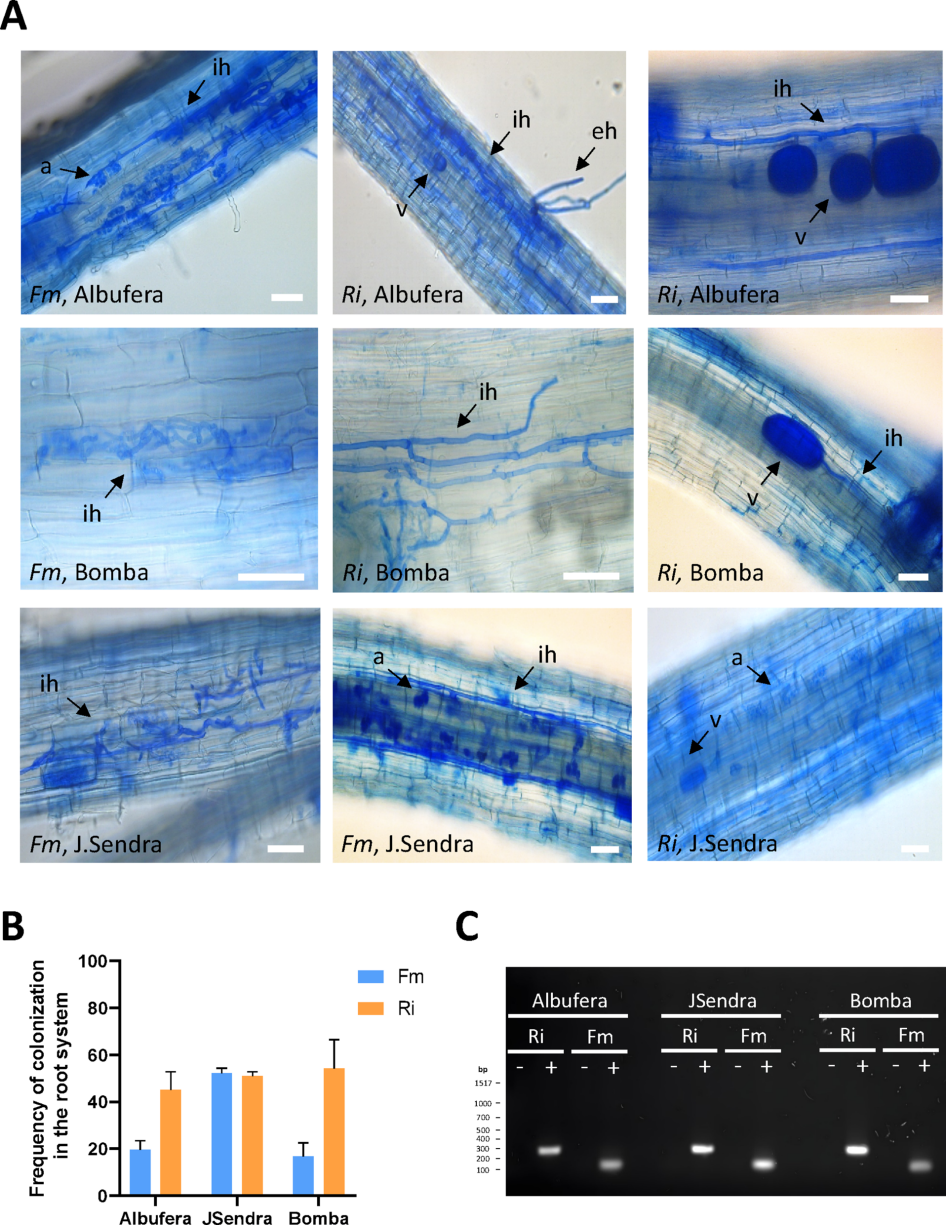
Root colonization by *R. irregularis* and *F. mosseae* in *japonica* rice varieties. Microscopic examination of cotton blue-stained roots of Albufera, Bomba, JSendra seedlings grown under aerobic conditions in nurseries was carried out at 10 weeks after AM inoculation. **A** Examples of AM fungal structures used as indicators of root colonization by the AM fungus, *R. irregularis* (Ri) or *F. mosseae* (Fm): extraradical hyphae (eh), intracellular hyphae (ih), arbuscules (a) and vesicles (v). Representative images are shown. Scale bar = 50 μm. **B** Frequency (F%) of colonization in roots of nursery seedlings at 10 weeks post-inoculation with either *R. irregularis* or *F. mosseae* according to the Trouvelot method. **C** PCR amplification of the 28 S rRNA gene from *R. irregularis*, and the small ribosomal subunit RNA gene from *F. mosseae*. +, AM-inoculated; -, mock-inoculated

Three rice cultivars were selected for this study, Albufera, Bomba and JSendra. For rice nursery preparation, seedlings were inoculated with either *R. irregularis* or *F. mosseae*, or mock-inoculated, and aerobically grown on soilless media used in greenhouse cultivation (e.g., 66.7% quartz sand, 0.3–0.8 mm; 22.2% turface; 11.1% vermiculite). Traditionally, root colonization by AM fungi is evaluated by microscopic observations of stained roots for detection of fungal morphological structures. In our study, root colonization by the AM fungus was assessed at different times after inoculation with one or another fungus (e.g., 5 weeks and 10 weeks). At 5 weeks after AM inoculation, fungal structures were barely detected by microscopic observations of cotton blue-stained roots of nursery-grown rice seedlings. By 10 weeks post-inoculation, however, the typical fungal structures were clearly observed root cortical root cells, such as arbuscules (the site in which active nutrient exchange between the two partners occurs), extraradical and intraradical hyphae and vesicles (Fig. 4A). The frequency of root colonization (F%) by *R. irregularis* was similar in all three cultivars (45.2%, 54.2% and 51.0% in Albufera, Bomba and JSendra, respectively). In *F. mosseae*-inoculated plants, however, the frequency of root colonization was lower in Albufera and Bomba than in JSendra (Fig. 4B). PCR analysis using AM-specific primers followed by DNA sequencing confirmed specific detection of both AM fungal species (*R. irregularis*, *F. mosseae*) in roots, whereas no amplification products were detected in mock-inoculated plants (Fig. 4C). This study confirmed that the 3 rice varieties here examined are susceptible to root colonization by the AM fungi *R. irregularis* and *F. mosseae*.

### Blast Resistance in Mycorrhizal Rice Plants in Low Fertility Flooded Fields

Field trials were then conducted with Albufera, Bomba and JSendra cultivars. For this, the rice seedlings were inoculated with *R. irregularis*, or mock-inoculated, and grown for 5 weeks in a nursery prior transplanting into paddy fields. As previously mentioned, by this time, the root cortex was poorly colonized by the AM fungus. However, we reasoned that 5 weeks of growth in the nursery would be enough time to ensure penetration of the fungus into the rice roots thus allowing functioning of the symbiotic relationship in flooded conditions.

In the field, the fungus *M. oryzae* can affect different above ground tissues of the rice plant, including leaves, nodes, necks and panicles. The incidence and severity of rice blast increases under high relative humidity and temperature. Field trials were conducted in two geographical regions in years 2023 and 2024, focusing on resistance to infection by the rice blast fungus *M. oryzae* and grain yield. For blast resistance, field trials surveyed leaf blast, node blast, neck blast and panicle blast. Seedlings of Albufera, Bomba and JSendra were inoculated with *R. irregularis*, or mock-inoculated, grown in nurseries for 5 weeks, and then manually transplanted into flooded fields.

Blast incidence varied in rice fields located in one or another rice-growing region, Valencia and Ebro delta, as well as among the two years (2023, 2024). For instance, panicle blast incidence was very low in Valencia fields in 2023, whereas leaf blast had a low incidence in Ebro delta fields in 2024. Considering all field trials (e.g., experiments in the two locations in the 2023 and 2024 cropping seasons), we found a positive effect of inoculation with *R. irregularis* on blast incidence in the three rice cultivars.

In Valencia, incidence of leaf blast was more severe in 2024 than in 2023, but SES values for leaf blast consistently decreased in AM-inoculated plants compared to the corresponding mock-inoculated plants (Tables 2 and 2023-Valencia). In 2023, SES scores in mock-inoculated Albufera, Bomba and JSendra plants were 4, 5 and 3, respectively, whereas in the corresponding AM-inoculated plants, SES scores were 3, 2 and 2, respectively (Tables 2 and 2023-Valencia). Also in Valencia, in 2024, SES values of 3 (Albufera), 7 (Bomba) and 4 (JSendra) were recorded in mock-inoculated plants, these values decreasing to 2 (Albufera), 6 (Bomba) and 3 (JSendra) in mycorrhizal plants. Representative images of leaf blast lesions in mycorrhizal and non-mycorrhizal plants in field trials conducted in Valencia are presented in Fig. S5A. Due to the low incidence of leaf blast, SES values were not recorded in field trials conducted in Ebro delta fields in year 2024.

**Table 2:** Blast resistance in AM-inoculated *japonica* rice varieties

		2023			2024			
		Valencia			Valencia		Ebro river delta	
		Mock	*R*. irregularis		Mock	*R*. irregularis	Mock	*R*. irregularis
Leaf blast (SES)	Albufera	4	3		3	2	Low incidence	
	Bomba	5	2		7	6		
	JSendra	3	2		4	3		
Neck blast (%)	Albufera	15.0 ± 2.89	5.0 ± 2.89^a^		3.33 ± 1.67	2.5 ± 1.44	2.5 ± 0	1.67 ± 0.83
	Bomba	10.0 ± 0	6.67 ± 4.41		6.67 ± 3.33	2.5 ± 1.44	1.67 ± 0.83	0.0 ± 0
	JSendra	18.33 ± 1.67	5.0 ± 0*		11.67 ± 1.67	3.33 ± 1.67*	5.0 ± 1.44	4.17 ± 1.67
Node blast (%)	Albufera	8.33 ± 3.33	3.33 ± 1.67		18.05 ± 1.82	14.23 ± 3.22	10.4 ± 1.62	2.97 ± 1.5*
	Bomba	23.33 ± 1.67	8.33 ± 4.41^a^		12.7 ± 1.58	5.68 ± 1.23*	n.d.	n.d.
	JSendra	15.0 ± 2.89	6.67 ± 1.67^a^		16.1 ± 1.82	8.5 ± 1.48*	10.52 ± 0.49	4.5 ± 0.79**
Panicle blast (%) (25% of lesions)	Albufera	Low incidence			8.33 ± 1.67	3.33 ± 1.67	20.0 ± 2.5	9.17 ± 2.20*
	Bomba				13.33 ± 4.41	6.67 ± 1.67	20.83 ± 2.2	18.33 ± 1.67
	JSendra				10.0 ± 2.89	5.0 ± 2.89	10.0 ± 2.5	2.5 ± 1.44^a^

Resistance to infection by the rice blast fungus under natural infection conditions in *japonica* rice varieties that have been inoculated with the AM fungus *R. irregularis*, grown in a nursery, and then transplanted to flooded fields. Experiments were conducted in rice fields located in Valencia (2023 and 2024) and Ebro delta (2024) during the rice cropping season (May-September). Blast incidence was evaluated at the harvest time. For each variety, 3 independent plots were designed for each condition (AM-inoculated, mock-inoculated), each plot containing 30 plants. In each experimental field, the three varieties were randomly distributed. Severity of leaf blast was assessed using the SES scoring system from IRRI. For evaluation of neck blast and panicle blast, 40 panicles and nodes were randomly harvested from plots for each variety and condition (3 plots/each condition and variety). Node blast was evaluated in 6 plants randomly harvested from each plot. Data represent mean ± SEM. Statistically significant differences were determined by Student’s t-test (^a^, *P* < 0.1; *, *P* < 0.05; **, *P* < 0.01). n.d., not determined

Inoculation with *R. irregularis* also conferred protection against neck blast (2023 and 2024), the positive effect of AM inoculation on neck blast being more evident in Valencia fields (Table 2). Of the three varieties, JSendra consistently exhibited better protection levels than Albufera and Bomba. Regarding node blast, Bomba was the most benefited variety from AM inoculation in Valencia in the two years (2023, 2024) (Table 2). In the field trials conducted in Valencia, representative images illustrating neck and node blast lesions in mycorrhizal and non-mycorrhizal plants are shown in Fig. S5B and Fig. S5C. Finally, in 2024, AM inoculation reduced panicle blast severity in all three varieties in both locations (Table 2; panicle blast levels were low in Valencia fields in 2023).

Together, results obtained in field trials demonstrated that inoculation with the AM fungus *R. irregularis* confers protection against the rice blast fungus. The magnitude of reduction of disease symptoms was dependent on the rice variety and the rice tissue affected (e.g., leaves, neck, node and panicle).

### Inoculation with an AM Fungus Increases Grain Yield of ***japonica*** Rice in Low Fertility Flooded Fields

We investigated whether inoculation with *R. irregularis* has an effect on grain yield in field-grown rice varieties. Productivity was surveyed in field trials conducted in 2023 and 2024 in Valencia), and 2024 in Ebro delta with the three rice cultivars, Albufera, Bomba and JSendra.

Panicles were harvested manually from each independent plot in these field trials. In both locations (Valencia, Ebro Delta), the highest yield was obtained on rice plants that have been inoculated with *R. irregularis* compared to mock-inoculated plants in 2023 and 2024 (Table 3). In Albufera, the percentage of increase in grain yield in AM-inoculated plants ranged from 10.84 to 11.3% compared to mock-inoculated plants: Valencia, 10.84% (2023) and 11.3% (2024); Ebro delta, 11.11% (2024) (Table 3). Grain yields in Bomba plants were also higher in *R. irregularis*-inoculated plants than in mock-inoculated plants, with increases of 5.67% (Valencia, 2023), 10.48% (Valencia, 2024), and 11.18% (Ebro delta, 2024). In JSendra, AM-inoculation increased grain yield by 13.93% (Valencia, 2023), 11.09% (Valencia, 2024) and 11.79% (Ebro delta, 2024) (Table 3).

**Table 3:** Grain yield in AM-inoculated *japonica* rice cultivars

		2023				2024							
		Valencia				Valencia				Ebro river delta			
		Mock	*R*. irregularis	Increase (%)		Mock	*R*. irregularis	Increase (%)		Mock	*R*. irregularis	Increase (%)	
Grain yield (g/plant)	Albufera	14.59 ± 2.87	16.17 ± 1.4	10.84		23.5 ± 0.97	26.57 ± 1.7	11.3		19.59 ± 1.26	21.76 ± 0.88	11.11	
	Bomba	9.91 ± 0.81	10.47 ± 0.92	5.67		20.39 ± 0.25	21.37 ± 0.47	10.48		20.42 ± 1.26	22.83 ± 1.98	11.18	
	JSendra	17.8 ± 1.61	20.28 ± 4.5	13.93		23.96 ± 0.48	26.56 ± 0.25*	11.09		21.94 ± 0.64	25.88 ± 0.55*	11.79	
Germination ratio (%)	Albufera	n.d.	n.d.	n.d.		81.07 ± 1.3	83.92 ± 1.3	2.85		84.36 ± 4.25	87.15 ± 0.95	2.8	
	Bomba	n.d.	n.d.	n.d.		86.22 ± 1.6	91.98 ± 2.02^a^	5.76		93.3 ± 0.77	96.35 ± 0.9 ^a^	3.06	
	JSendra	n.d.	n.d.	n.d.		75.01 ± 1.14	77.33 ± 0.16	2.77		92.44 ± 0.44	95.11 ± 0.44*	2.67	

Albufera, Bomba and JSendra seedlings were inoculated with *R. irregularis*, grown in a nursery, and transplanted into low fertility flooded fields. Percentage of germination of seeds collected from mycorrhizal and non-mycorrhizal plants is also presented. The rice plants were grown in Valencia (2023, 2024) and Ebro delta (2024) from May to September. For each variety 3 independent plots were designed for each condition (mycorrhizal, non-mycorrhizal), each plot containing 30 plants for each variety and condition. In each experimental field, the three varieties were randomly distributed. Data represent the mean ± SEM. Statistically significant differences were determined by Student’s t-test (^a^, *P* < 0.1; *, *P* < 0.05). n.d., not determined

Finally, germination tests were carried out with seeds harvested from mycorrhizal and non-mycorrhizal plants in field trials conducted in Valencia and Ebro delta in the 2024 cropping season. The percentage of seeds that germinated during a time intervale of 5 days was consistently higher in mycorrhizal plants relative to non-mycorrhizal plants (Table 3). This increase was observed in the three rice varieties, in field trials performed in the two geographical regions. The percentage of germination in seeds from mycorrhizal Albufera plants increased by 2.85% and 2.80% in Valencia and Ebro delta, respectively, relative to non-mycorrhizal plants (Table 3). Germinability in seeds from mycorrhizal Bomba plants increased by 5.76% and 3.06% in Valencia and Ebro delta, respectively, whereas in JSendra, the percentage of germinated seeds harvested from mycorrhizal plants increased 2.67% (Ebro delta) and 2.77% (Valencia) (Table 3).

Collectively, results obtained in field trials in Valencia and Ebro delta in 2023 and 2024 revealed that inoculation with the AM fungus *R. irregularis* and aerobic growth in nurseries followed by transplanting to flooded fields positively impacts rice production and seed germinability, at least in the three cultivars assayed in this study (Albufera, Bomba, JSendra).

## Discussion

This study provides evidence that inoculation with an AM fungus protects temperate *japonica* rice against the blast fungus when grown in natural, low fertility soil both under controlled and field conditions, while increasing grain yield in field-grown plants (flooded rice). In this study, commercial formulations of AM fungi (*R. irregularis*, *F. mosseae*) were used for inoculation of rice seedlings. The AM-inoculated seedlings were initially grown under aerobic conditions in nurseries and then transplanted to conventional paddy fields. Growth and Pi nutrition increased in AM-inoculated rice varieties grown in natural, low fertility soil, this effect being observed with both fungi, either *R. irregularis* or *F. mosseae*.

It is well known that the genotype of the two partners, host plant and AM fungus, can determine the outcome of the interaction. In agroecosystems, diverse environmental factors might also influence AM fungal effects on plant traits, including soil composition and properties, geographical features, and climate conditions. Our analyses clearly show that AM fungi confer blast protection in rice plants under controlled and field conditions. Furthermore, protection against leaf blast, neck blast, node blast and panicle blast was observed in *R. irregularis*-inoculated rice plants in field trials conducted in two different geographical regions and two consecutive years. These observations established a solid basis for the causal link between presence of the AM fungus (either *R. irregularis* or *F. mosseae*) and blast resistance. Different possibilities, not exclusive between them, can be considered to explain positive effects of AM inoculation on blast disease. On the one hand, improved nutrition in mycorrhizal rice plants might improve plant fitness which, in turn, might enhance the plant’s ability to cope with pathogen infection. On the other hand, the AM symbiosis might potentiate the plant’s capability to develop effective defense responses to pathogen infection. While this study did not explicitly addressed mechanisms underlying defense responses in mycorrhizal rice plants during *M. oryzae* infection, we previously reported that inoculation with *R. irregularis* (or *F. mosseae*) in aerobically grown rice plants (greenhouse conditions) promotes the systemic induction of defense-related genes in leaves of mycorrhizal rice plants, a process that resembles mycorrhiza-induced resistance (MIR) (Campos-Soriano et al. 2012; Campo and San Segundo 2020; Guigard et al. 2023). Both effects, nutritional status improvement and MIR might well contribute to blast resistance in field-grown mycorrhizal rice plants.

In the literature, there are examples of varietal differences in plant responses to AM fungi in different crops, including rice (Pérez-López et al. 2013; Campos et al. 2018; Davidson et al. 2019; Campo et al. 2020; Berger and Gutjahr 2021; Guigard et al. 2023). It is also true that most studies so far carried out to assess plant mycorrhizal responses used inert substrates for the AM-inoculated plants growth (e.g., sand/turface/vermiculite mixture) under greenhouse conditions. In the present study, all the rice/AM fungus combinations stimulated plant growth, increased leaf Pi content, and conferred blast resistance in plants grown on natural soil under controlled conditions and field conditions. These findings differ from those previously found in AM-inoculated rice cultivars growing on inert substrate, where AM inoculation induced positive, neutral and even negative effects on rice growth and disease resistance (Campo et al. 2020; Guigard et al. 2023). Together, these observations underscore the importance of using natural soil in studies on mycorrhizal rice plants where the overall ecosystem, for instance, microbial communities present in the soil, might be crucial in determining the outcome of the rice/AM fungus interaction. Previous studies carried out in a Rice Diversity Panel representing different subpopulations of rice (*indica*, *tropical japonica*, *temperate japonica*, *aus* and *aromatic*) also revealed differences among cultivars in terms of responsiveness to inoculation with *R. irregularis* (e.g., growth stimulation) (Davidson et al. 2019). Here, it is important to highlight that rice cultivars assayed in those studies were genetically more distant among them than cultivars investigated in the present work. In other studies, variable responses to AM inoculation between *japonica* and *indica* rice cultivars (e.g., growth phenotype) were described, the *japonica* cultivars responding better to mycorrhization than *indica* cultivars (Guigard et al. 2023). Contrary to this, differences between rice genotypes to *F. mosseae* were found not to be related to *indica* or *japonica* origins (Suzuki et al. 2015). Presumably, genetic diversity among rice cultivars might determine differences in host mycorrhizal responsiveness, these differences being more important in distantly related rice varieties. In line with this, variable mycorrhizal responses among wheat cultivars were reported (Mao et al. 2014; García de León et al. 2020; Thirkell et al. 2022). Here, ancient wheat cultivars (e.g., cultivars developed before 1950) were found to benefit most from the AM symbiosis than cultivars released after 1950 when the use of fertilizers was adopted for optimal crop yields (Hetrick et al. 1993). Although rice is one of the most important food crops in the world, few research focused on the host genetics in the rice/AM fungi interaction. In addition to plant genetic factors, environmental conditions are likely of major importance for variations in the level of root colonization among rice genotypes (Berger and Gutjahr 2021).

Furthermore, the mechanistic and molecular mechanisms influencing genetic variability in AM responsiveness in rice plants have not been fully elucidated yet. The first genetic polymorphism determining quantitative root colonization by AM fungi in rice was the LysM receptor-like kinase CERK1, required for the perception of short-chain chitin oligomers (He et al. 2019). The receptor kinase CERK1 has been reported to play a dual role in symbiosis and immunity signalling (Zhang et al. 2015; Carotenuto et al. 2017). Also, genome wide association mapping for root colonization by AM fungi identified some putative quantitative trait loci (QTLs) contributing to that variation (Davidson et al. 2019). Further investigation is then required to shed light on genetic factors and molecular mechanisms contributing to variation in root colonization by AM fungi in rice, and how this variation might affect agronomical traits of interest. On the other hand, molecular mechanisms underlying the AM symbiosis have been widely studied in the model legumes *Medicago truncatula* and *Lotus japonicus*. However, cautions must be taken with translating results from dicotyledonous species into monocotyledonous crops. The reason is that root colonization by AM fungi in dicotyledonous and monocotyledonous species appears to be governed by different factors (Gutjahr et al. 2008; Mukherjee and Ané 2010; Jacott et al. 2017).

Current practices in rice cultivation rely on the use of fertilizers, while modern rice cultivars are under intense in-breeding programs for the development of high-yielding varieties. As evaluation of agronomical traits in newly developed rice varieties is usually carried out in fertilized fields, these varieties might have lost the ability to establish mycorrhizal associations. Rice varieties more responsive to AM fungi should therefore be identified to better exploit potential benefits received from the AM symbiosis. Furthermore, successive interbreeding steps are commonly used in breeding programs for the development of locally adapted rice cultivars. This fact is exemplified by some of the rice varieties assayed in this work. Bomba was the ancestor of Albufera, whereas JSendra was the ancestor of Argila and Regina. Apart from Bomba, which is a landrace genetically distant from the rest of the varieties used in this study, Senia is a common ancestor to varying degrees of Argila, Albufera, Baixet and JSendra. Recently, the population structure and genetic distance in a collection of rice varieties, including Spanish varieties, has been reported, this study demonstrating the relationship between Spanish rice varieties (García-Romeral et al. 2024). A reduction in the gene pool and genetic polymorphisms during successive inbreeding processes might well explain similar phenotypic responses in the panel of rice cultivars investigated in this work (e.g., growth stimulation and Pi nutrition, blast resistance and productivity). Increasing genetic diversity in modern rice cultivars is expected to help in identifying rice/AM fungus interactions benefiting most from AM fungi. Future studies should also concentrate on determining the symbiotic compatibility in wild rice and ancient cultivated rice varieties. The knowledge gained on these studies is fundamental to understand how the breeding history used for generation of modern rice cultivars might have affected the AM symbiosis in rice.

Not only the genetic background and breeding history of the rice plant, but also the type of soil in which the plants are grown and other environmental factors might affect the establishment and development of the AM symbiosis in rice. Results obtained in soilless-grown plants (e.g., substrates used in greenhouse cultivation) are not necessarily representative of environmental conditions relevant for agriculture. Soil properties and microbiota (e.g., phosphate solubilizing microorganisms) (Svenningsen et al. 2018; Sweeney et al. 2021) might well affect rice/AM fungi interactions. For this reason, in this work, low fertility soil collected from rice fields were not subjected to sterilization. It is then tempting to hypothesize that microorganisms in the soil, such as Pi solubilizing bacteria, might facilitate Pi acquisition by plant roots, a situation that does not occur on plants grown on soilless substrate (usually sterilized substrate). Soil microorganisms present in the soil assayed in this work might contribute to mobilization of nutrients, making nutrients more readily available to the plant, an aspect that deserves further investigation.

One consideration in this research is that the effect of flooding is expected to depend on the extent to which the AM fungus has penetrated the root system when flooding occurs. Results presented in this study indicated that a period of 5 weeks of growth in a nursery was enough to allow the rice plant to benefit from the effects of AM inoculation after transplanting into flooded fields. We hypothesized that the positive effects of inoculation with an AM fungus might be the consequence of AM-mediated improvement of Pi nutrition. The observed reduction in blast disease in AM-inoculated, field-grown rice plants might also contribute, at least in part, to improve grain yields.

## Conclusions

Results here presented support the potential of the AM symbiosis to benefit the rice plant under flooded conditions for the development of sustainable production systems in rice. From a practical point of view, the application of AM fungi in nurseries may be integrated with conventional rice cultivation systems in paddy fields while contributing to reduce water use compared to continuous flooded conditions. Positive responses to inoculation with either *R. irregularis* or *F. mosseae* were found in all the *japonica* rice cultivars investigated in this work. As the benefits received by the AM symbiosis are strongly context-dependent, the effect of AM inoculation on agronomical traits needs to be evaluated on a case-by-case basis in different geographical areas. The impact of the AM symbiosis on blast resistance (e.g., leaf blast, neck blast, node blast, and panicle blast) might also vary depending on the rice genotype and geographical location. Results here presented set the basis for further research to elucidate the exact mechanisms by which the AM symbiosis benefits rice, e.g., temperate *japonica* rice, in flooded field conditions. However, a better understanding of endogenous and exogenous factors controlling the AM symbiosis in rice is still needed to better exploit the benefits of the AM symbiosis in developing sustainable production systems in rice cultivation.

## Electronic Supplementary Material

Below is the link to the electronic supplementary material.

Supplementary Material 1. Fig. S1 Location of the rice-growing regions in eastern Spain in Valencia and Ebro River delta. Soils used in this study, low fertility and high fertility soil, were harvested from rice fields located at the Ebro River delta site. Fig. S2 Experimental design followed to grow rice plants in the nursery for field experiments. Fig. S3 Experimental design used in field assays conducted in Valencia (2023 and 2024) and Ebro river delta (2023). Distribution of plots containing mock-inoculated or R. irregularis-inoculated plants of the rice varieties Albufera, Bomba and JSendra. Fig. S4 Pi content of rice plants that have been grown on soil collected from rice fields, low fertility soil or high fertility soil. Fig. S5 Typical symptoms of leaf blast, neck blast, node blast in field-grown rice plants.

## Data Availability

No datasets were generated or analysed during the current study.
