# Supplementary material for: Arbuscular Mycorrhizal Fungi Increase Blast Resistance and Grain Yield in Japonica Rice Cultivars in Flooded Fields
Source: Rice (N Y). 2025 May 30;18:47. doi: 10.1186/s12284-025-00805-4 (PMC12125454; doi:10.1186/s12284-025-00805-4)
Supplement: Supplementary file 1 — Supplementary Material 1. Fig. S1 Location of the rice-growing regions in eastern Spain in Valencia and Ebro River delta. Soils used in this study, low fertility and high fertility soil, were harvested from rice fields located at the Ebro River delta site. Fig. S2 Experimental design followed to grow rice plants in the nursery for field experiments. Fig. S3 Experimental design used in field assays conducted in Valencia (2023 and 2024) and Ebro river delta (2023). Distribution of plots containing mock-inoculated or R. irregularis-inoculated plants of the rice varieties Albufera, Bomba and JSendra. Fig. S4 Pi content of rice plants that have been grown on soil collected from rice fields, low fertility soil or high fertility soil. Fig. S5 Typical symptoms of leaf blast, neck blast, node blast in field-grown rice plants. [file 12284_2025_805_MOESM1_ESM.docx]

**Supplemental Material**

**Arbuscular mycorrhizal fungi increase blast resistance and grain yield in *japonica* rice cultivars in flooded fields**

Héctor Martín-Cardoso, Laia Castillo, Iratxe Busturia, Gerrit Bücker, Luís Marqués, Eva Pla, Mar Català-Forner, Concha Domingo, and Blanca San Segundo

**Fig. S1** Location of the rice-growing regions in eastern Spain in Valencia and Ebro river delta. Soils used in this study, low fertility and high fertility soil, were harvested from rice fields located at the Ebro River delta site. **A** Map showing the location of the rice fields in the Ebro river delta (violet circle) and Valencia (orange square). **B** Experimental design used in field experiments conducted in Valencia and Ebro river delta. Field trials were carried out in the cropping seasons of 2023 (Valencia), and 2024 (Valencia and Ebro river delta). Rice was grown from May to September (2023 and 2024 in Valencia; 2023 in Ebro River delta).

**Fig. S2** Experimental design followed to grow rice plants in the nursery for field experiments. **A** Design of field trials. 10-day-old seedlings were inoculated with *R. irregularis*, or mock-inoculated, and aerobically grown for 5 weeks in seedling beds (35 cells/tray, 350 ml/cell). Plant height and leaf Pi content were determined. A representative image is shown. Then, the rice seedlings were transplanted to paddy fields. **B** Blast disease assays under controlled conditions. 10-day-old seedlings were inoculated with either *R. irregularis* or *F. mosseae*, or mock-inoculated, grown in nurseries for 4 weeks as in A (seedling beds), and spray-inoculated with a suspension of *M. oryzae* spores (5 x 10^5^ spores/ml). Blast disease was evaluated at 1 week post-inoculation with *M. oryzae* spores. **C.** Analysis of root colonization by an AM fungus. 10-day-old seedlings were inoculated with either *R. irregularis* of *F. mosseae* and grown in the nursery (160 ml-cones). Root colonization by AM fungi was evaluated at 5 weeks or 10 weeks post-inoculation with the AM fungus. A representative image at 10 weeks post-inoculation is shown.

**Fig. S3** Experimental design used in field assays conducted in Valencia (2023 and 2024) and Ebro river delta (2023). Distribution of plots containing mock-inoculated or *R. irregularis*-inoculated plants of the rice varieties Albufera (A), Bomba (B) and JSendra (JS).

**Fig. S4** Pi content of rice plants that have been grown on soil collected from rice fields, low fertility soil or high fertility soil. Plants were grown in one or another type of soil for 4 weeks. Bars represent mean ± SEM of 3 biological replicates, each one from a pool of 3 plants. Statistically significant differences were determined by Student’s t-test (**, P < 0.01; ***, P < 0.001). FW, fresh weight.

**Fig. S5** Typical symptoms of leaf blast (A), neck blast (B), node blast (C) in field-grown rice plants.
